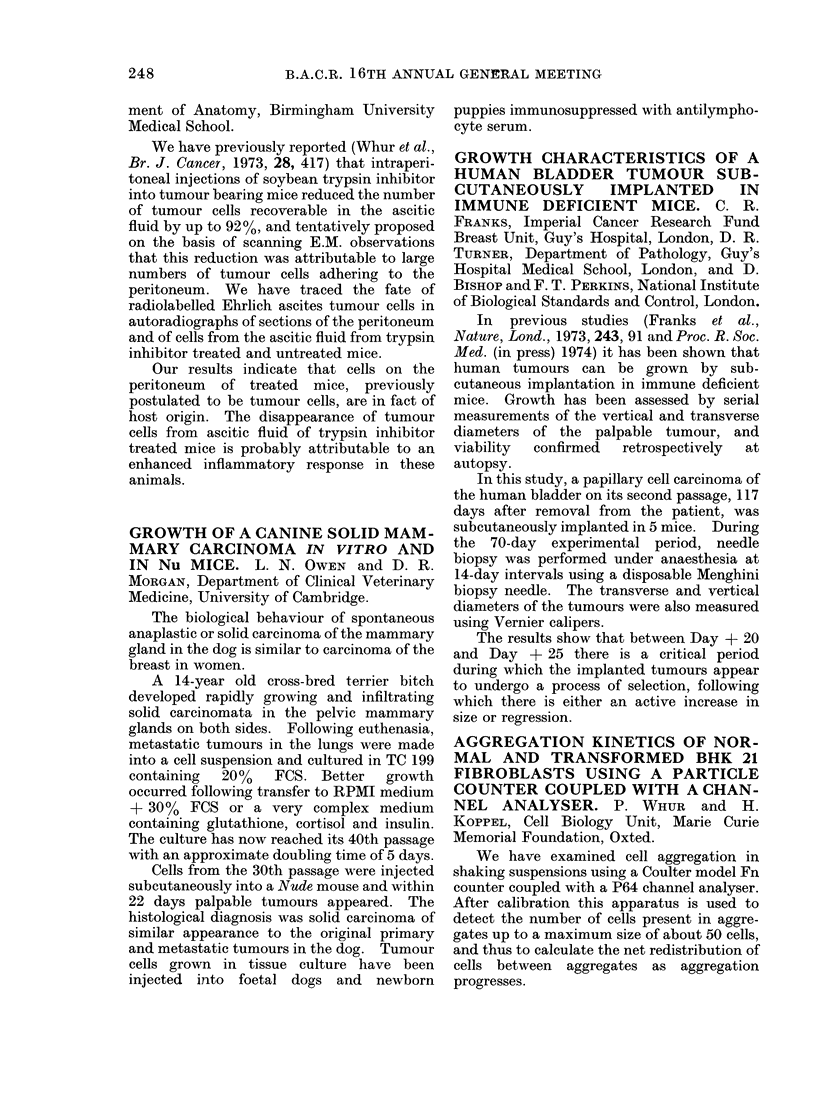# Proceedings: Growth characteristics of a human bladder tumour subcutaneously implanted in immune deficient mice.

**DOI:** 10.1038/bjc.1975.182

**Published:** 1975-08

**Authors:** C. R. Franks, D. R. Turner, D. Bishop, F. T. Perkins


**GROWTH CHARACTERISTICS OF A HUMAN BLADDER TUMOUR SUBCUTANEOUSLY IMPLANTED IN IMMUNE DEFICIENT MICE.** C. R. FRANKS, Imperial Cancer Research Fund Breast Unit, Guy's Hospital, London, D. R. TURNER, Department of Pathology, Guy's Hospital Medical School, London, and D. BISHOP and F. T. PERKINS, National Institute of Biological Standards and Control, London.

In previous studies (Franks *et al.*, *Nature, Lond.*, 1973, **243**, 91 and *Proc. R. Soc. Med.* (in press) 1974) it has been shown that human tumours can be grown by subcutaneous implantation in immune deficient mice. Growth has been assessed by serial measurements of the vertical and transverse diameters of the palpable tumour, and viability confirmed retrospectively at autopsy.

In this study, a papillary cell carcinoma of the human bladder on its second passage, 117 days after removal from the patient, was subcutaneously implanted in 5 mice. During the 70-day experimental period, needle biopsy was performed under anaesthesia at 14-day intervals using a disposable Menghini biopsy needle. The transverse and vertical diameters of the tumours were also measured using Vernier calipers.

The results show that between Day + 20 and Day + 25 there is a critical period during which the implanted tumours appear to undergo a process of selection, following which there is either an active increase in size or regression.